# The effect of comorbidity on mortality in elderly patients undergoing emergency abdominal surgery: a systematic review and metaanalysis

**DOI:** 10.3906/sag-2001-27

**Published:** 2021-02-26

**Authors:** Fadime ÇINAR, Göknur PARLAK, Fatma ETİ ASLAN

**Affiliations:** 1 Department of Health Management, Faculty of Health Sciences, Sabahattin Zaim University, İstanbul Turkey; 2 Department of Nursing, Institute of Graduate Studies, Bahçeşehir University, İstanbul Turkey; 3 Department of Nursing, Faculty of Health Sciences, Bahçeşehir University, İstanbul Turkey

**Keywords:** Emergency, abdominal surgery, elderly, mortality, comorbid

## Abstract

**Background/aim:**

With the increase in the elderly population, the elderly proportion needing emergency surgery is also increasing. Despite medical advances in surgery and anesthesia, negative postoperative outcomes and high mortality rates are still present in elderly patients undergoing emergency surgery. Comorbidities are described as the main determining factors in poor outcomes. In this metaanalysis, it was aimed to investigate the effect of comorbidity on mortality in elderly patients undergoing emergency abdominal surgery.

**Materials and methods:**

The studies published between 2010-2019 were scanned from databases of Google Scholar, Cinahl, Pub Med, Medline and Web of Science. Quality criteria proposed by Polit and Beck were used in the evaluation of the included studies. Interrater agreement was calculated by using the Kappa statistic, effect size by using the odds ratio, and heterogeneity among studies by using the Cochran’s Q statistics. Kendall’s Tau-b coefficient and funnel plot were used to determine publication bias.

**Results:**

A total of 9 studies were included in the research. There was a total of 1330 cases in the studies. The total mortality rate was 21% (n = 279), the total rate of having a comorbid factor was 83.6% (n = 1112), and the rate of having a comorbid factor in mortality was 89.2% (n = 249). According to the fixed effects model, the total effect size of comorbid factors on causing mortality was not statistically significant with a value of 1.296 (C.I; 0.84-1.97; P > 0.05).

**Conclusion:**

Our study revealed that comorbidity had no significant effect on causing mortality in geriatric patients undergoing emergency abdominal surgery. There are controversial results in the literature, and in order to reach more precise results, studies involving wider groups of patients and further studies examining the specific effect of certain comorbid conditions are needed.

## 1. Introduction

The number of the elderly population is increasing year by year in both developed and developing countries due to the increase in the world population and developments in the diagnosis and treatment of chronic health problems. The number of people over the age of 65 in the world is estimated to increase to 1.5 billion by 2050 [1,2]. In Turkey, the population over the age of 65 has increased by 16% between 2014 and 2018, from 6,192,962 to 7,186,204, and the ratio of the elderly population to the general population is 8.3%. This figure is estimated to reach 10% by 20251Demographic İnformation (2018). Turkey [online]. Website http://www.tuik.gov.tr/UstMenu.do?metod=temelist/ , [accessed 01.12.2019]..

The increase of life expectancy at birth increases the proportion of the elderly population and the need for emergency surgery in this age group [3,4]. This group of patients accounts for approximately 50% of all emergency surgeries and 75% of postoperative mortality [3]. It is reported that in 2040, 24% of the world’s population will consist of elderly individuals, and half of this population is likely to undergo surgery. The conditions requiring emergency surgery in elderly individuals are gallbladder and biliary tract emergencies, acute bowel obstruction, hernia, and peptic ulcer perforation [5,6]. 

The symptoms of the disease differ due to decreased physiological capacity and changes in the body’s metabolic and endocrine response in the cases of acute abdomen in patients aged 65 years and over, and these often lead to late diagnosis and more complicated progression of the perioperative phase [6]. It is a known fact that the rate of morbidity and mortality due to surgical intervention increases with ageing [7]. Moreover, the rate of having comorbid factors increases with age. A plethora of studies show that the primary risk factor for poor surgical outcome in the elderly is comorbidity rather than chronological age [8]. In elderly patients undergoing emergency surgery, the presence of physiological disorders such as renal failure, sepsis or shock and comorbidities increase mortality more, compared to elective surgery [6,9].

A geriatric population is a group where comorbid factors are more common and more serious, and it is important to determine the possible effects of the presence of comorbid conditions on patient prognosis and mortality in this population increasing every day. There are many studies conducted in this field in the literature, and these studies report different results. This study aimed to determine the effect of comorbid factors on mortality in elderly patients undergoing emergency abdominal surgery.

## 2. Materials and methods

### 2.1. Study selection 

Medline, Google Scholar, Web of Science, PubMed and Cinahl databases were scanned for the words “emergency” and “abdominal” and “surgery” and “geriatrics” or “elderly” and “mortality” and “comorbidity” registered in MeSH and their Turkish equivalents. The articles published between 2010 and 2019 were selected. Inclusion criteria were used to determine the articles to be included in this study: Articles that meet the requirements 1) studies involving 65-year-old and older patient group, 2) the sample of the studies consisting of patients who had undergone emergency abdominal surgery for nontraumatic reasons, 3) studies examining the relationship between mortality and comorbid factors, 4) sample size being appropriate, 5) being an original study, 6) being able to reach the full text of the research were evaluated within the scope of the study. 

### 2.2. Data quality assessment 

**Figure 1:**
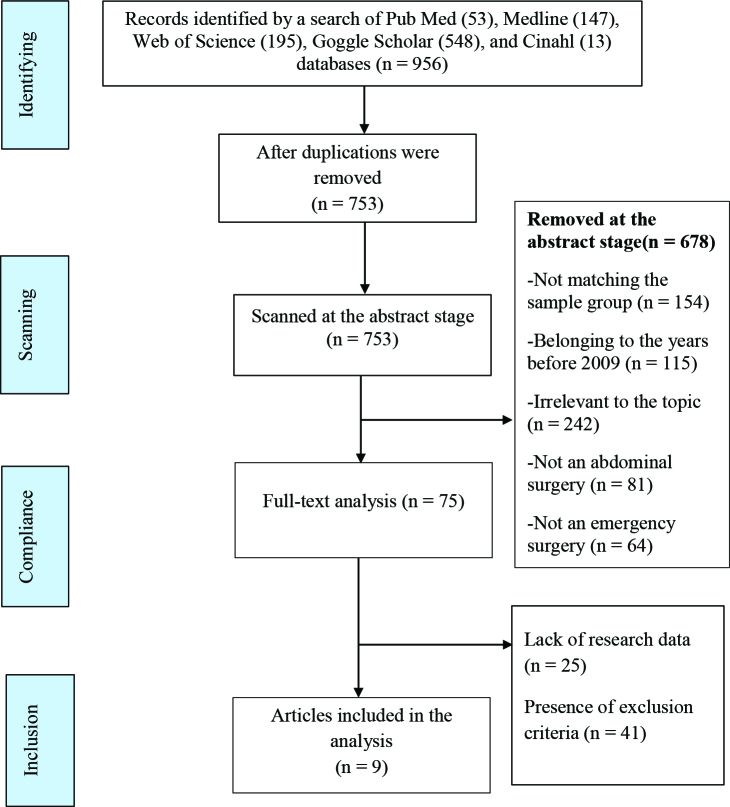
PRISMA 2 (preferred reporting items for systematic reviews and metaanalyses statement).

The full texts of 75 studies selected by the two researchers (GP, FÇ) according to the title, abstract, and eligibility criteria, respectively, were evaluated independently, and the studies (n = 9) eligible for the analysis were selected for metaanalysis. The stages of the study selection process are shown in PRISMA 2 (preferred reporting items for systematic reviews and meta-analysis statements) flow diagram in Figure 1 [10]. A total of 12 of the criteria for assessing data quality in quantitative studies proposed by Polit and Beck were used in the evaluation of the studies included in the metaanalysis. The studies were evaluated by three researchers on all criteria separately, and a value of “1 point” was given if it met each item fully, and a value of “0 point” was given if it did not. In total, articles with a score of 0-4 were rated as articles with poor quality, articles with a score of 5-9 were rated as articles with moderate quality, and articles with a score of 9-12 were rated as articles with strong quality [11].

### 2.3. Statistical analysis 

PRISMA (preferred reporting items for systematic reviews and metaanalyses statement) and MOOSE (metaanalysis of observational studies in epidemiology) criteria were used in the preparation of the metaanalysis [12]. 

In the research, CMA 3 (comprehensive metaanalysis version 3) was used for the implementation of metaanalysis technique, and Kappa statistic was used for inter-rater reliability.

The “odds ratio” was used in the calculation of effect size in this research. Odds ratio being equal to 1 means that there is no relationship between variables, while the odds ratio above 1 indicates that the risk ratio had an effect [13]. 

Cochran’s Q statistic was used to test heterogeneity between studies in this research [14]. Apart from Cochran’s Q test, the coefficients I2 were used to calculate heterogeneity [15]. In heterogeneity assessment, I2 is considered to be none if it is below 25%, low if it is 25%-50%, moderate if it is 51%-75%, and high if it is above 75% [16]. Since heterogeneity I2 was low in the study, fixed-effects model was used in metaanalysis. In this study, P < 0.05 was considered statistically significant, and the results of the research were interpreted according to these values. 

In the first phase, the funnel plot was examined to determine whether there was a publication bias in the metaanalysis studies. Another way to determine publication bias in metaanalysis research is to calculate Kendall’s Tau-b coefficient. In the absence of publication bias, this coefficient is expected to be close to 1 and the two-tailed P-value is not expected to make a significant difference, i.e. the P-value is expected to be greater than 0.05 [13]. 

## 3. Results 

Nine studies investigating the relationship between postoperative mortality and comorbid factors in geriatric patients undergoing emergency abdominal surgery were found.

### 3.1. Study characteristics

Details of the 9 studies included are summarized in Table. One of the studies included in the metaanalysis was prospective research type and eight were retrospective research types. The number of patients in these studies published between 2010 and 2018 ranged from 72 to 430. There was a total of 1330 cases in the studies. The mean age of these cases was 81.82 years, the total mortality rate was 21% (n = 279), the total comorbid factor was 83.6% (n = 1112), and the rate of having a comorbid factor in mortality was 89.2% (n = 249). 

**Table:** The details of the study.

Author/Country	Sample	Objective of the study	Age /Type of surgery	Mortality rate (%)	Comorbidity cases (%)	Conclusion	Quality score
Özkan et al.Turkey-2010	92	To report on the experience of emergency abdominal surgery in elderly patients and to identify the risk factors affecting mortality in these patients.	73.3/Abd.	15.2	81.5	The presence of an underlying chronic condition has a negative effect on prognosis in patients undergoing emergency surgery, resulting in greater mortality rates than in elective surgery cases.	9.3
Mirbagheri et al.Australia-2010	179	To examine the results of abdominal surgery in patients aged 85 and over in terms of mortality, morbidity and changes in care status and to analyze the factors predicting outcomes.	88.6/Abd.	22.6	100	According to the study, comorbidity in the elderly is not a significant predictor of mortality in emergency abdominal surgery	10.3
Ukkonen et al.Finland-2015	430	To examine recovery as a result of emergency abdominal surgery in elderly patients with acute abdominal pain.	76/Abd.	14.2	26.3	The absence of comorbidity did not reduce the mortality rate statistically (p=0.341).	10.3
Wilson et al.England-2014	73	To determine the risk factors predicting in-hospital morbidity and mortality among elderly patients undergoing emergency abdominal surgery.	84/Abd.	38	100	The presence of COPD has been found to significantly increase the risk of in-hospital mortality after surgery.	9.3
Mzoughı et al.Tunisia-2018	100	To determine the factors predicting mortality after emergency abdominal surgery in the elderly.	77/Abd.	50	72	In the study, comorbidities were not found to be associated with mortality.	10.6
Merani et al.Canada-2014	170	To examine surgical outcomes, including identifying factors associated with in-hospital mortality and morbidity by separating very old patients who have undergone emergency general surgery.	84/Abd.	14.7	91	There is no clear correlation between the number of comorbidities and postoperative outcomes (morbidity or mortality).	10.6
Fukuda et al.Japan-2012	94	To report on the experience of emergency abdominal surgery in elderly patients and to identify the risk factors affecting mortality in these patients.	85.6/ Abd.	16	75.5	Comorbidity is not a significant prognostic factor for older patients with emergency abdominal surgery.	9.3
Racz et al.England-2012	145	To determine the outcomes of elective surgery and emergency abdominal surgery in patients aged 90 years and older, and to evaluate the performance of POSSUM and p-POSSUM systems as predictors of mortality.	91/Abd.	15	100	Heart failure and other cardiac comorbidities may predict mortality in patients aged 90 years and older.	9.3
Kenig et al.Poland-2015	184	To determine the prognostic role of comorbidities in patients admitted to the emergency department and underwent abdominal surgery.	76.9/ Abd.	45	84.2	Myocardial insufficiency, vascular disease, lipid disorders, and kidney disease are risk factors for 30-day mortality.	9.3

POSSUM: the physiologic and operative severity score for the enumeration of mortality and morbidity; p-POSSUM: Portsmouth POSSUM; COPD: chronic obstructive pulmonary disease; Abd.: Abdomen.

### 3.2. Statistical analysis results 

Of the studies (n = 9) evaluated by independent evaluators, 5 were evaluated to have a “moderate” quality, and 4 to have a “strong” quality. A total of 9 studies met this criterion and were included in the metaanalysis, as studies evaluated to have strong and moderate quality would be included in the metaanalysis. The mean score of the 9 studies included in the metaanalysis as a result of the quality assessment is given in Table.

In this study, kappa values ranged between 0.721-0.838 based on interrater reliability compliance analysis of articles. The overall fit ratio was 0.779, and it was found to be significantly fit. Kappa value < 0: worse than that expected by chance; 0.21-0.40: poor; 0.41-0.60: moderate; 0.61-0.80: good; and 0.81-1.00: very good level of agreement [17]. The kappa value of 0.77 in this study indicates that there is a good agreement between encoders.

### 3.3. Effect magnitudes and heterogeneity 

According to the findings obtained from the research studies, the sample of the study consists of 1330 people. In this study, the heterogeneity test of the comorbidity variable, which is considered a risk factor after emergency abdominal surgery, has been applied.

As a result of heterogeneity test, P-value was greater than 0.05 and it was determined that the studies examined according to the comorbidity variable had a homogeneous structure. Since heterogeneity was low in the study (I2 = 0%), fixed-effects model was used in metaanalysis. 

**Figure 2:**
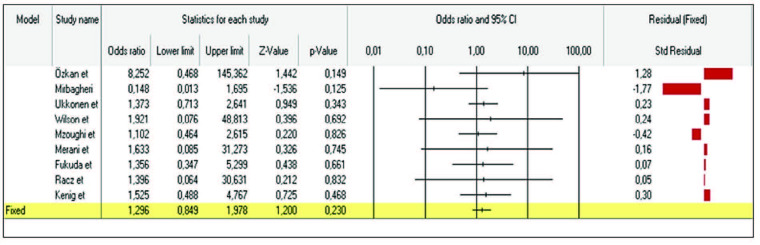
Forest graph of the effect of comorbidity on mortality.

In Figure 2, metaanalysis results of the 9 studies examining the risk factors involved in causing mortality in geriatric patients undergoing emergency abdominal surgery were shown with forest plot. The analysis based on the fixed effects model revealed that the overall effect size of comorbid factors on causing mortality was not statistically significant with a value of 1.296 (C.I; 0.84-1.97; P > 0.05).

Four of the studies [2,4,18,19] we included in our study examined the effect of the type of surgery on mortality. The statistical analysis of these studies examined the effect of commonly used types of surgery on mortality. We found the overall effect size of the type of surgery on mortality as 1.277 (CI; 0.87-1.25; P > 0.05) with the random-effects model, revealing that this effect size of the surgery type was not statistically significant to affect mortality.

### 3.4. Analysis of broadcast bias 

**Figure 3:**
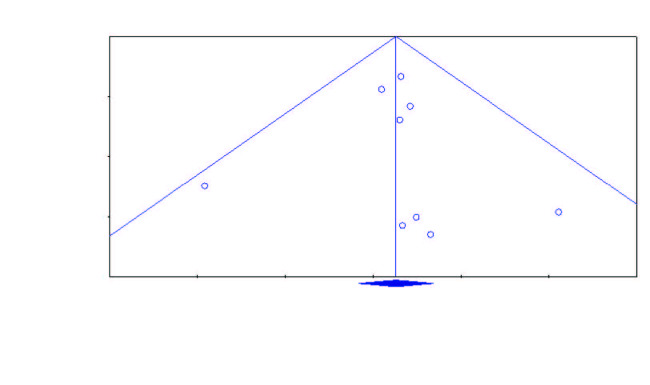
Funnel plot.

The results of the funnel plot, which is also considered as a visual summary of the metaanalysis dataset and shows the probability of publication bias, are shown in Figure 3. As shown in Figure 3, the majority of the 9 studies included in the research are positioned very close to the combined effect size and in the upper parts. Another way to determine publication bias in metaanalysis research is to calculate Kendall’s Tau-b coefficient. According to the values calculated in the statistics (Kendall’s Tau-b = 0.05; P = 0.83), publication bias was not detected in the studies included in the metaanalysis. 

## 4. Discussion

Our study reveals the results of mortality and comorbid factors in the population aged 65 years and older after emergency abdominal surgery. Total 30-day mortality rates were in the range of 14% to 38% in the studies examined using the metaanalysis. This result is consistent with the mortality rate reported in the literature, which ranges from 11% to 37% [6]. While the risk of 30-day mortality after emergency abdominal surgery is approximately 25% for individuals aged 80 years and over, this rate approaches 50% for individuals aged 90 years and over [9]. 

The decrease in renal, pulmonary reserves and the presence of comorbid factors in elderly individuals negatively affects prognosis after emergency surgery and increases mortality more than elective surgery [6]. As a result of the systematic review of nine studies, we observed that the presence of comorbid conditions had no significant effect on mortality in geriatric patients undergoing emergency abdominal surgery. It was revealed that the cases of geriatric emergency abdominal surgery (n = 1330) included as a result of the study had at least one comorbid factor, with a high rate of 89.2%. In addition, 89.2% (n = 249) of the 279 cases resulting in death were also found to have comorbid factors. 

Four of the studies [2,3,4,8] evaluated within the scope of the metaanalysis reported findings that some specific diseases (such as heart and kidney failure, chronic obstructive pulmonary disease) increased mortality, whereas five studies [18-22] reported that comorbid factors had no effect on mortality in elderly patients undergoing emergency abdominal surgery. In our study, the overall effect score was P > 0.05, and it was confirmed that comorbid factor had no effect on mortality in elderly patients undergoing emergency abdominal surgery. 

Some authors report that certain types of surgery are risk factors for mortality in old individuals [18]. Several studies are available in the literature, reporting that surgery for mesenteric ischemia, peptic ulcer perforation [2,6,19], and intestinal obstruction [4,6] are associated with high mortality. Of the studies we included in our study, four of them [2,4,18,19] examined the effect of the type of surgery on mortality. In the analysis with the random-effects model on these studies, it was found that the overall effect size of the surgical type on mortality was not statistically significant with a calculated value of 1.277 (CI; 0.87-1.25; P > 0.05). One of the studies additionally examined the relationship between the surgeon’s experience and mortality [19]. We suggest that the effects of the type of surgery and the surgeon’s experience on mortality should be addressed and examined further in future studies.

Estimating mortality for emergency surgery in older individuals may help in determining expectations for families and clinicians or may affect the surgical intervention decision [9]. It is extremely important that the health personnel plan and manage the process and care correctly in cooperation with both the patient’s family and relatives. 

The small number of studies included was the most important limitation of this metaanalysis. In our metaanalysis, the presence of comorbidity was examined in general. Although no significant effect was detected, different findings can be seen in studies that examine the specific effects of comorbidities separately. Differences within the elderly population (65-80 and 80 years old or older) may also have affected the results.

In conclusion, the metaanalysis of the studies conducted between 2010 and 2019 shows that comorbidity alone has no significant effect on the mortality of elderly patients undergoing emergency abdominal surgery. It is considered that the type of comorbidity (dysthymia, heart failure, renal failure, chronic obstructive pulmonary disease) is more important in terms of postoperative prognosis than the number or presence of comorbidity. Considering the different information on the subject in the literature, this suggests the need for further analysis involving studies including groups of patients with larger sampling and characteristics and examining the effect of specific comorbid conditions on mortality to reach more precise conclusions. 
